# Protein Tyrosine Nitration in Plant Nitric Oxide Signaling

**DOI:** 10.3389/fpls.2022.859374

**Published:** 2022-03-11

**Authors:** José León

**Affiliations:** Instituto de Biología Molecular y Celular de Plantas (Consejo Superior de Investigaciones Científicas – Universidad Politécnica de Valencia), Valencia, Spain

**Keywords:** nitration, nitric oxide, 3-nitro-tyrosine, post-translational modification, sensing, signaling

## Abstract

Nitric oxide (NO), which is ubiquitously present in living organisms, regulates many developmental and stress-activated processes in plants. Regulatory effects exerted by NO lies mostly in its chemical reactivity as a free radical. Proteins are main targets of NO action as several amino acids can undergo NO-related post-translational modifications (PTMs) that include mainly S-nitrosylation of cysteine, and nitration of tyrosine and tryptophan. This review is focused on the role of protein tyrosine nitration on NO signaling, making emphasis on the production of NO and peroxynitrite, which is the main physiological nitrating agent; the main metabolic and signaling pathways targeted by protein nitration; and the past, present, and future of methodological and strategic approaches to study this PTM. Available information on identification of nitrated plant proteins, the corresponding nitration sites, and the functional effects on the modified proteins will be summarized. However, due to the low proportion of *in vivo* nitrated peptides and their inherent instability, the identification of nitration sites by proteomic analyses is a difficult task. Artificial nitration procedures are likely not the best strategy for nitration site identification due to the lack of specificity. An alternative to get artificial site-specific nitration comes from the application of genetic code expansion technologies based on the use of orthogonal aminoacyl-tRNA synthetase/tRNA pairs engineered for specific noncanonical amino acids. This strategy permits the programmable site-specific installation of genetically encoded 3-nitrotyrosine sites in proteins expressed in *Escherichia coli*, thus allowing the study of the effects of specific site nitration on protein structure and function.

## Introduction

Plant developmental programs as well as responses to stress involve a wide variety of processes affecting gene expression that include transcription, post-transcriptional processing, and alternative splicing, translation, and post-translational modifications (PTMs; recently reviewed in Zhang et al., 2021). After translation, proteins are often targets of a wide array of PTMs that may alter their 3D structure, cofactor- or ligand-binding, stability to proteolysis, or intracellular localization, and therefore, their function and fate. PTMs are thus potential modifiers of protein function that when occurring in response to environmental or endogenous cues and affect to signaling proteins have potential in regulating signal transduction pathways. Despite the variety of responses triggered by different stresses, a major common response involves the production of reactive oxygen species (ROS), nitric oxide (NO), and other reactive nitrogen species (RNS), and reactive sulfur species (RSS; Del Río, 2015; Choudhury et al., 2017; De Gara and Foyer, 2017; Hancock, 2019). The combined action of NO, ROS, and RNS regulate many developmental and stress-related processes in plants including pollen and seed germination, elongation of shoots and roots, root architecture, stomatal closure and related abiotic stresses, leaf greening, mitochondrial function, several developmental transitions including flowering or senescence, and cell death. Many of these regulatory functions are exerted directly on varied protein targets that undergo different PTMs such as carbonylation (Tola et al., 2021); sulfenylation and persulfidation of cysteines (Aroca et al., 2021; Wang et al., 2021); methionine sulfoxidation (Drazic and Winter, 2014); S-nitrosylation (Lindermayr et al., 2005); and tyrosine nitration (Bottari, 2015; Kolbert et al., 2017; Arasimowicz-Jelonek and Floryszak-Wieczorek, 2019), phosphorylation at Ser, Thr, or Tyr residues (Gow et al., 1996; Kong et al., 1996); sumoylation (Miura et al., 2009; Niu et al., 2019); and polyubiquitination (Walsh and Sadanandom, 2014). Many of these PTMs are reversible but some of them, such as carbonylation and tyrosine nitration are irreversible, and frequently associated to protein inactivation and degradation. Among PTMs, S-nitrosylation and nitration are linked to the production of NO and often involved in signaling processes downstream NO sensing (Astier and Lindermayr, 2012).

## Nitric Oxide Biosynthesis and Sensing

**Figure 1:**
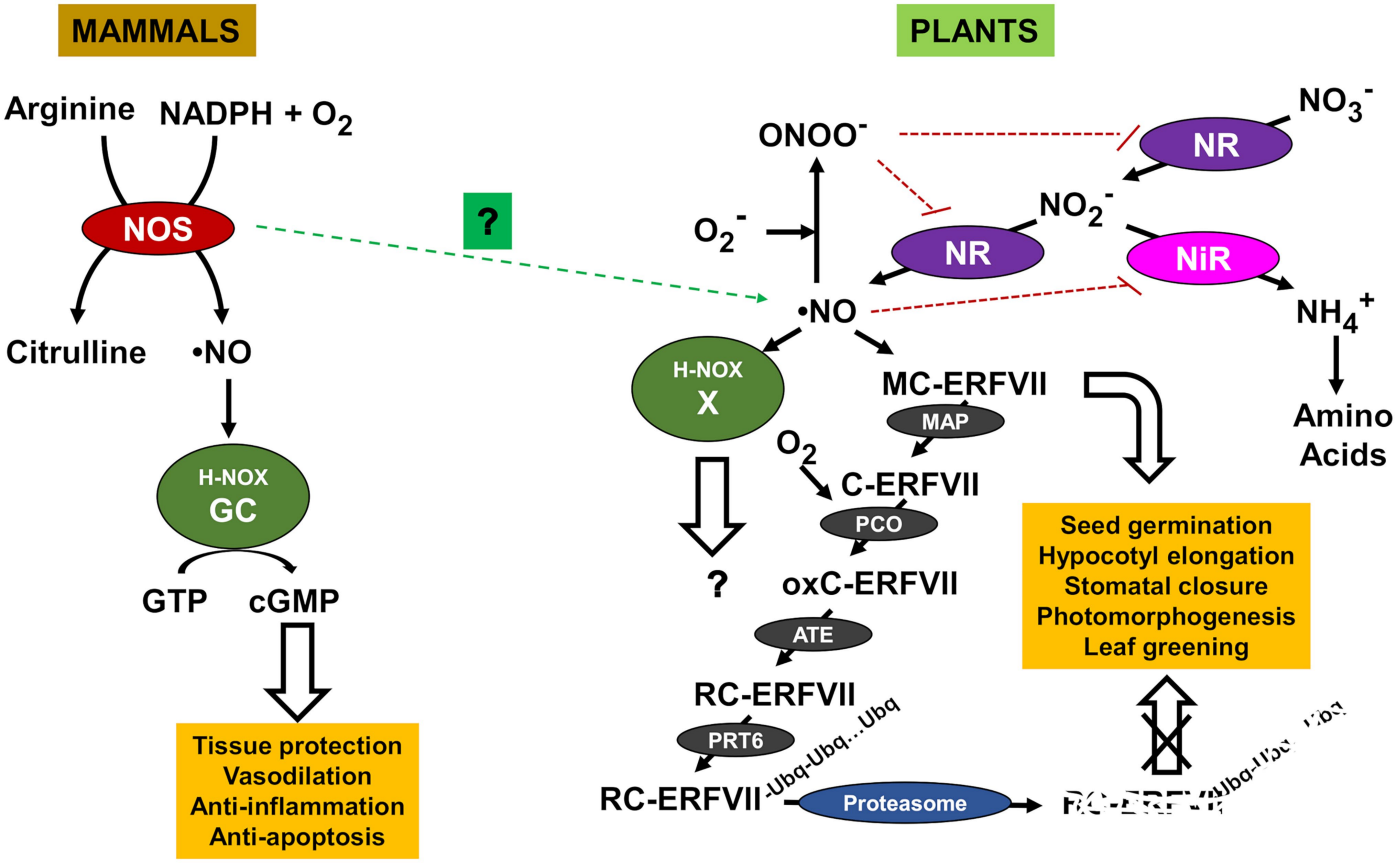
Nitric oxide (NO) production and sensing pathways in mammals and plants. Arginyl transferase (ATE), group VII Ethylene Response Factor (ERFVII), Cyclic guanosine monophosphate (cGMP), guanosine triphosphate (GTP), guanylate cyclase (GC), heme NO/oxygen motif (H-NOX), methionine aminopeptidase (MAP), nitrate reductase (NR), nitrite reductase (NiR), NO synthase (NOS), oxidized cysteine ERFVII (oxC-ERFVII), peroxynitrite (ONOO^−^), plant cysteine oxidase (PCO), E3 ubiquitin ligase Proteolysis6 (PRT6), arginylated cysteine ERFVII (RC-ERFVII), and unknown NO sensor (X). NO- and peroxinitrite-triggered inactivation are shown by blunt-ended red dotted lines. Still uncertain involvement of NOS in NO production is shown with green dotted arrow.

Nitric oxide is a gas molecule ubiquitously present in all living organisms, from prokaryotes to eukaryotes, that is involved in the regulation of a wide array of biological processes from development to stress-activated responses (Hancock, 2020). NO and their target processes have been extensively documented in humans, other mammals, and vertebrates, invertebrates, plants, low eukaryotic organisms such as protozoan and yeast, and bacteria (Torreilles, 2001). NO is endogenously synthesized through different pathways depending on the organism. Since early 90s, it is well-known that NO is produced through the oxidation of the reduced nitrogen molecule arginine, catalyzed by NO synthases (NOS) in animals (Marletta, 1993; Moncada, 1993; Palmer, 1993). NOS are homodimer enzymes that utilize L-arginine as the substrate, molecular oxygen, and reduced nicotinamide-adenine-dinucleotide phosphate (NADPH) as co-substrates, and flavin adenine dinucleotide (FAD), flavin mononucleotide (FMN), and (6R-) 5,6,7,8-tetrahydro-L-biopterin (BH4) as cofactors (Förstermann and Sessa, 2012). Many prokaryotes also generate NO through arginine-dependent NOS-like systems (Adak et al., 2002; Sudhamsu and Crane, 2009). However, unlike mammalian NO synthases, bacterial NO synthases do not contain a reductase domain (Filippovich, 2010). Although NOS activity has been measured in yeast and plants, no NOS ortholog has been identified to date. NO synthesis and sensing in plants differ from animals, and many controversial issues remain unsolved (León and Costa-Broseta, 2020). To date, no such NOS enzymes have been identified in plants (Jeandroz et al., 2016), though a NOS protein has been identified in the marine green alga *Ostreococcus tauri* (Foresi et al., 2010, 2015). Therefore, although NOS activity has been reported in plants it remains unclear whether this oxidative pathway operates (Kolbert et al., 2019). Instead, plants synthesize NO under normoxic conditions mostly from the oxidized nitrogen molecule nitrate through two sequential reduction steps to nitrite and further to NO catalyzed by nitrate reductase (NR), thus as a branch of the nitrate assimilatory pathway (Lozano-Juste and León, 2010; Astier et al., 2018; León and Costa-Broseta, 2020). NO-related differences between organisms are not restricted to their biosynthetic pathways but also apply to the way is sensed and signaled. Animal and early prokaryotes share structural similarity in NO-sensing proteins with a heme-containing domain that enables NO sensing by the heme NO/oxygen (H-NOX) motif. In mammals, NO is sensed through soluble guanylate cyclase (GC) receptor that transduces the initial NO signal to the secondary messenger cyclic guanosine monophosphate (cGMP; Arnold et al., 1977; Zhao et al., 1999). Effectors, such as cGMP-dependent protein kinases, ion channels, and phosphodiesterases are downstream regulated by cGMP, then controlling varied physiological processes such as regulation of blood vessel dilatation, immune function, and neurotransmission in the brain and peripheral nervous system. Although soluble GCs have been identified both in plants and yeast (Kuo et al., 1998), none of them have the biochemical features enabling their functions as NO sensors. Unlike animals, plants do not mediate NO signaling through the classical NO/cGMP signaling module, suggesting that the evolution of nitric oxide signaling diverged between animal and green lineages (Astier et al., 2019). The way NO is sensed in plants differs from that reported for mammals as they lack a specific receptor. However, it has been recently reported that plant proteins not related to GCs but containing the H-NOX motif might be involved in NO sensing, thus proposing the existence of an evolutionary conserved heme-based NO sensor (Wong et al., 2021). Future work will address whether these H-NOX motif-containing proteins are true NO sensors and, if they are, whether they are organ-, tissue-, or process-specific or instead behave as general plant NO sensors. Nevertheless, a NO sensing mechanism not related to H-NOX motif has been demonstrated to act as a plant NO sensor in plants. Many NO-regulated physiological processes in plants are controlled through a proteolytic mechanism determining the fate of several regulatory proteins. This proteolytic pathway depends on the N-terminal sequence of the target proteins that constitutes a so-called N-degron. Proteins containing N-degrons are specifically recognized by E3 ubiquitin (UBQ) ligases called recognins that polyubiquitinate them. The Cys/Arg branch of N-degron pathway acts on proteins containing a Cys in position 2, which must be oxidized in a process requiring both O_2_ and NO and catalyzed by Plant Cysteine Oxidases (PCO). After the subsequent N-terminal arginylation, substrates are further polyubiquitinated. The group VII of ethylene response factors (ERFVIIs) are substrates of this pathway and are polyubiquitinated by the recognin Proteolysis6 (PRT6) that prepares them for the proteolytic degradation by the 26S proteasome. This process has been characterized as a NO sensor controlling multiple physiological processes including seed germination, seedling establishment, hypocotyl elongation, stomata aperture, apical hook opening, and leaf greening in *Arabidopsis* (Gibbs et al., 2014; Abbas et al., 2015). Moreover, such mechanism has been also characterized as a general sensor of abiotic stresses and pathogen responses (Vicente et al., 2017, 2019). However, it remains controversial whether the NO essential requirement for ERFVII degradation operates at Cys2 oxidation step or later during ERFVII arginylation or polyubiquitination. In yeast, the expression of *Arabidopsis* PCOs seems to be sufficient to trigger the N-terminal cysteine oxidation, thus pointing to the dispensability of nitric oxide at this step (Puerta et al., 2019). Nevertheless, it must be assessed whether this also happens in plants, in which some PCO encoding genes are induced by NO (Castillo et al., 2018). A comparative scheme showing the differences between NO biosynthesis and sensing in mammals and plants is shown in Figure 1. Nevertheless, the N-degron-mediated regulation of ERFVII levels is crucial to control many NO-related processes as it determines the available transcription factor able to transactivate a wide array of secondary regulatory proteins, including ABI5 and other hormone-related transcription factors that finally regulate multiple developmental and stress-related responses (Figure 1).

## Signaling Downstream no Sensing Involves Diverse Post-translational Modifications Including Protein Nitration

Nitric oxide signaling downstream ERFVII-mediated sensing is highly dependent on the levels and function of these transcription factors. ERFVIIs regulate different target processes that includes responses to hypoxia (Gibbs et al., 2011; Gasch et al., 2016; Loreti et al., 2016) and ABA-regulated seedling establishment (Zhang et al., 2018). Specific ERFVIIs such as RAP2.12 regulates carbon metabolism (Paul et al., 2016), and together with RAP2.3 negatively regulates NO biosynthesis and responses through a rheostat-like mechanism in *Arabidopsis* (León et al., 2020). Despite the numerous processes that have been reported to be regulated by NO through ERFVII stability, the mechanism underlying such regulatory functions is often unknown. In addition to the ERFVII-mediated NO signaling, the reactive nature of NO makes it to react with other molecules as a primary action. Such NO reaction with proteins ends with PTMs that include the S-nitrosylation of Cys (Gupta et al., 2020) and, upon previous reaction with superoxide to form peroxynitrite (Liaudet et al., 2009; Vandelle and Delledonne, 2011), the nitration of Tyr, Trp, and to a lesser extent also Phe and His (Sabadashka et al., 2021). Besides peroxynitrite, the •NO_2_ radical formed from nitrite through peroxidase-mediated oxidation under certain conditions is also an efficient nitrating agent of proteins (Jones, 2012). The effect of nitrating agents on proteins are somehow regulated through the action of diverse antioxidant systems that includes glutathione peroxidases (Sies et al., 1997) as well as the most relevant plant antioxidant systems such as glutathione, ascorbic acid, tocopherols, and flavonoids (Arasimowicz-Jelonek and Floryszak-Wieczorek, 2011). By using mass spectrometry (MS) techniques, many S-nitrosylated and nitrated plant proteins have been identified (Lozano-Juste et al., 2011; Feng et al., 2019). Moreover, several algorithms have been developed to predict the probability of identifying S-nitrosylation and nitration sites in target proteins (Kolbert and Lindermayr, 2021; Nilamyani et al., 2021). However, the *in vivo* identification of S-nitrosylation and nitration sites represents a difficult task, and the characterization of the effects of nitration and S-nitrosylation on protein function are significantly less studied. For protein nitration, these studies are mainly limited by the low proportion of *in vivo* nitrated peptides compared to non-modified ones, the unstable nature of nitro-derivatives that tend to be converted to amino-derivatives in reductive environments, and by the lack of specificity in artificial nitration systems used to increase the amount of nitrated peptides for further MS-based analysis.

**Figure 2:**
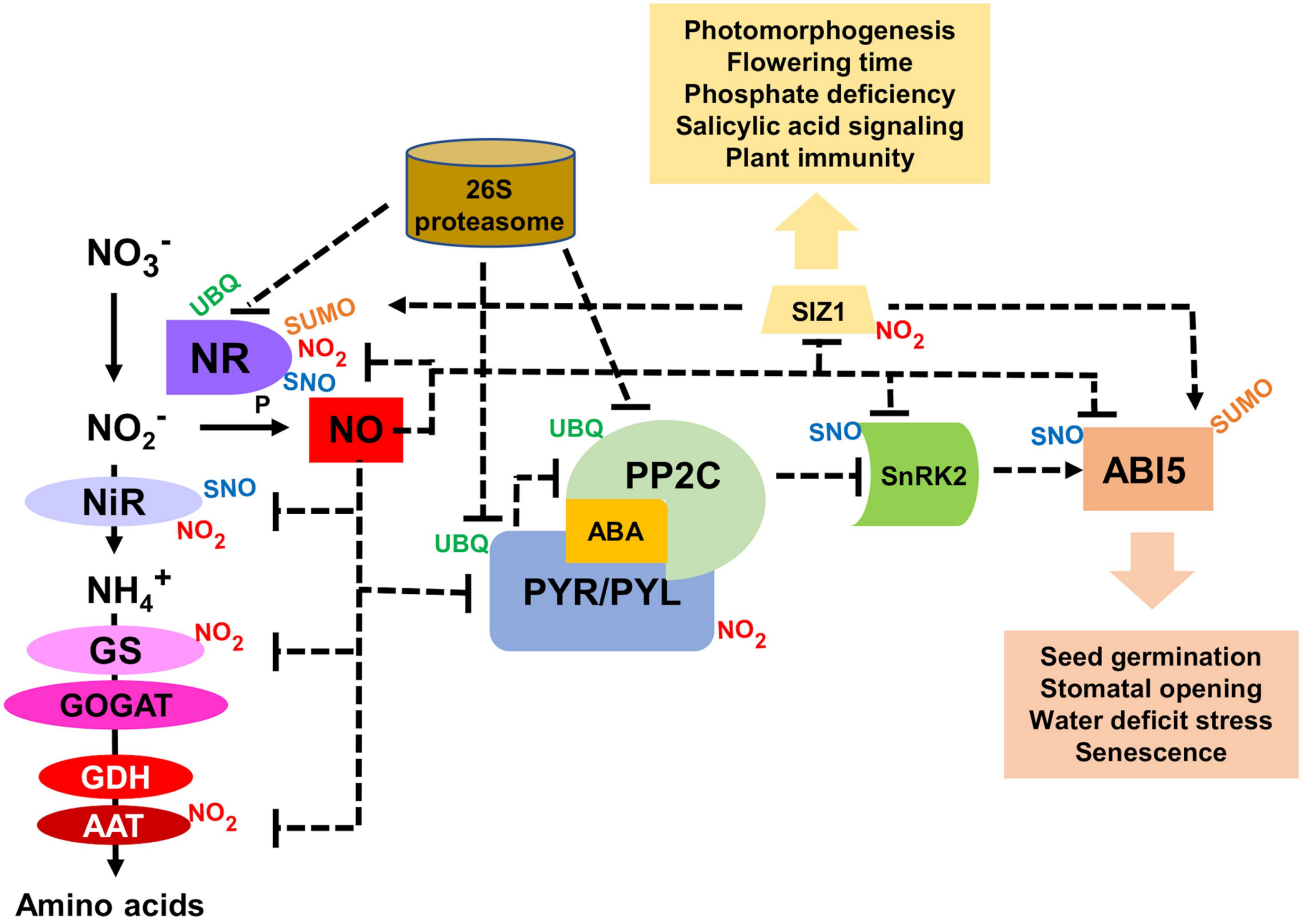
Functional interaction between NO and ABA signaling through NO-related post-translational modifications (PTMs). Aspartate aminotransferase (AAT), ABA-insensitive 5 (ABI5), glutamine synthetase (GS), glutamate dehydrogenase (GDH), glutamate synthase (GOGAT), nitrate reductase (NR), nitrite reductase (NiR), type 2C protein phosphatase (PP2C), Pyrabactin resistant ABA receptor (PYR), PYR-like (PYL), SAP and MIZ1 domain-containing ligase1 (SIZ1), sucrose non-fermenting1-related protein kinase 2 (SnRK2), small ubiquitin-like modifier (SUMO), nitrosothiol (SNO), and ubiquitin (UBQ). Dotted arrows and blun-ended lines represent activation and inactivation, respectively.

In contrast to Cys S-nitrosylated proteins, which can be readily reversed to unmodified protein by enzyme catalyzed processes (Benhar et al., 2009; Malik et al., 2011; Sengupta and Holmgren, 2013), Tyr-nitrated proteins were initially thought to be not reversible and ineluctably targeted for degradation (Souza et al., 2000; Zhang et al., 2019; Lee et al., 2020). However, it has been reported that animal cells have the capacity to denitrate Tyr-nitrated proteins, though the nature of the enzyme involved in denitration remains unclear (Gow et al., 1996; Kuo et al., 1999; Görg et al., 2007; Kang and Akbarali, 2008; Abello et al., 2009; Deeb et al., 2013; Ferrer-Sueta et al., 2018). A denitrating activity has not been reported yet in plants. Instead, a reversibility mechanism, which is slower than the enzyme-catalyzed denitration, could be based on the rapid protein degradation coupled to *de novo* protein synthesis. The protein nitration-induced proteolysis that has been reported in human cells (Souza et al., 2000; Zhang et al., 2019; Lee et al., 2020) has also been shown functionally relevant in the regulation of the ABA perception and nitrate-related NO synthesis in *Arabidopsis* (Castillo et al., 2015; Costa-Broseta et al., 2020, 2021). Nitration-induced proteasomal degradation of PYR/PYL/RCAR receptors have been reported as a potential rapid mechanism to control ABA signaling under certain conditions (Castillo et al., 2015). Moreover, NO also regulates ABA signaling through the S-nitrosylation and the subsequent KEEP ON GOING E3 ligase- and CULLIN4-mediated polyubiquitination of ABI5 transcription factor (Albertos et al., 2015). Besides, the function of ABI5 is modulated through sumoylation by the SUMO E3 ligase SIZ1 (Miura et al., 2009), which was previously identified as an *in vivo* nitration target (Lozano-Juste et al., 2011). It is worth mentioning that the NO biosynthetic enzyme NR has been reported to be relocated to nuclei by SIZ1 (Kim et al., 2018), thus potentially representing and additional link between NO and ABA signaling. SIZ1 plays also key regulation on salicylic acid (SA) signaling (Miura et al., 2010) and plant immunity (Gou et al., 2017; Niu et al., 2019), thus suggesting SIZ1 could be a relevant node in NO, ABA, and SA signaling through nitration, sumoylation, and polyubiquitination (Figure 2). Moreover, SIZ1 has also been recently characterized as an important regulator of many biological processes in plants including the repression of histone deacetylase and further regulation of flowering time (Gao et al., 2021), phosphate deficiency-related responses (Zheng and Liu, 2021), photomorphogenesis (Zhang et al., 2020), and cell wall formation (Liu et al., 2019). This could be a good example of the impact of Tyr nitration on a signaling node due to the regulatory relevance of SIZ1. However, more work is needed to explore the functional relevance of SIZ1 nitration, and specifically the identification of Tyr nitration sites will be essential for a better understanding on nitration-sumoylation functional interactions controlling multiple plant processes.

Nitric oxide also plays key roles in pathogen-triggered responses in plants (Jedelská et al., 2021), and under oxidative conditions, peroxynitrite is synthesized and functions as an effector of NO-mediated signaling (Alamillo and García-Olmedo, 2001; Saito et al., 2006; Vandelle and Delledonne, 2011). During the plant hypersensitive disease resistance response, protein tyrosine nitration has emerged as an important node between NO and ROS underlying a mechanism of co-operative interaction (Cecconi et al., 2009). Many plant-pathogen interactions are accompanied by the enhanced expression of Pathogenesis-Related (PR) proteins with diverse anti-pathogenic activities. Several PR proteins have been reported to be targets of Tyr nitration (Lozano-Juste et al., 2011; Tanou et al., 2012; Takahashi et al., 2016; Arasimowicz-Jelonek et al., 2016). Tyr nitration of proteins was also activated in tomato plants infected with leaf mold disease, though no specific targets have been identified (Chen et al., 2017). The effect of PR protein nitration on function/activity remains unknown and it should be further studied to know the functional relevance of this PTM on anti-pathogenic activity. Moreover, since PR proteins are frequently secreted to the apoplast, nitration may occur both at the cytoplasm and apoplast and when the modification takes place in the cytoplasm could also interfere with the final localization of these proteins. The involvement of protein nitration is not restricted to the interaction of plants with bacteria or fungi. Increased S-nitrosylation and nitration of proteins was detected during the infection of *Arabidopsis* roots with beet cyst nematodes (Labudda et al., 2020).

## Nitration of the Enzymes of Nitrate Assimilation and no Biosynthesis Represents a Potential Regulatory Loop

Nitrate together with ammonium are the most abundant nitrogen (N) source on Earth (Crawford and Glass, 1998). Both N sources present complex interactions that determine their transport/uptake, their allocation and assimilation (Hachiya and Sakakibara, 2017). Nitrate is photosynthetically assimilated by plants through a reductive linear pathway that converts nitrate into nitrite, and then to ammonium, requiring the sequential catalysis by NR and nitrite reductases (NiR; Campbell and Kinghorn, 1990). After reduction, ammonium is then incorporated to 2-oxoglutarate through the glutamine synthetase (GS)—glutamine oxoglutarate aminotransferase (GOGAT)/glutamate synthase metabolic cycle (Krapp, 2015) as well as the activities of aspartate aminotransferases (AATs), glutamate dehydrogenase (GDH), and asparagine synthetases (Kishorekumar et al., 2020). These enzymes enable the initial N incorporation to the amino acids Gln, Glu, Asn, and Asp, and then by transamination to the other amino acids. NRs, together with their main function catalyzing the conversion of nitrate to nitrite, can also reduce nitrite to NO (Maia and Moura, 2015; Chamizo-Ampudia et al., 2017), thus making NO a side product of nitrate assimilation. Most of the enzymes involved in nitrate assimilation have been identified as nitrated in Tyr residues. *Arabidopsis* NRs NIA1 and NIA2 as well as the only NiR1 enzyme has been recently reported to be Tyr-nitrated (Costa-Broseta et al., 2020, 2021). In *Arabidopsis* NRs, nitration occurs at two Tyr residues that coordinates flavin co-factor thus potentially hampering its efficient binding, and therefore the proper electron transfer flow throughout the enzyme (Costa-Broseta et al., 2021). For NiR1 nitration might not be the most relevant NO-triggered PTM, as it has been reported the S-nitrosylation of two Cys residues involved in coordinating the 4Fe-4S cluster and siroheme, which are key co-factors for the NiR redox activity of NiR1 (Costa-Broseta et al., 2020). Moreover, nitration not only affects to the first two enzymes of nitrate assimilation, but also GS has been identified as targets of nitration (Lozano-Juste et al., 2011). GS enzymes from mammals and fungi seem to be nitrated and inactivated, thus affecting the metabolism of Glu and ammonia in liver (Frieg et al., 2021) and the secondary metabolism in fungi (Zhu et al., 2021), respectively. Plant GDH seem to be involved mainly in Glu oxidation to supply cells with carbon skeletons under carbon limiting conditions (Robinson et al., 1991). Similarly, in mammals, GDH catalyzes the reversible oxidative deamination of Glu into 2-oxoglutarate and ammonium, which is a N source for the urea cycle. The enzyme from renal cortex cell mitochondria has been reported to be nitrated (Ishii et al., 2013). In turn, no reports on plant GDH nitration are available. Regarding other N metabolism-related enzymes, AATs catalyze the conversion of aspartate and 2-oxoglutarate to oxaloacetate and glutamate, thus playing mostly a transaminase function. The chloroplastic isoform from *Arabidopsis* was found nitrated *in vivo* (Lozano-Juste et al., 2011), and both the cytosolic and mitochondrial mammalian enzymes were inactivated upon nitration (Scandurra et al., 1975; DiCola et al., 1976), thus representing a potential regulatory target of NO in regulating the distribution of reduced N between amino acids.

**Figure 3:**
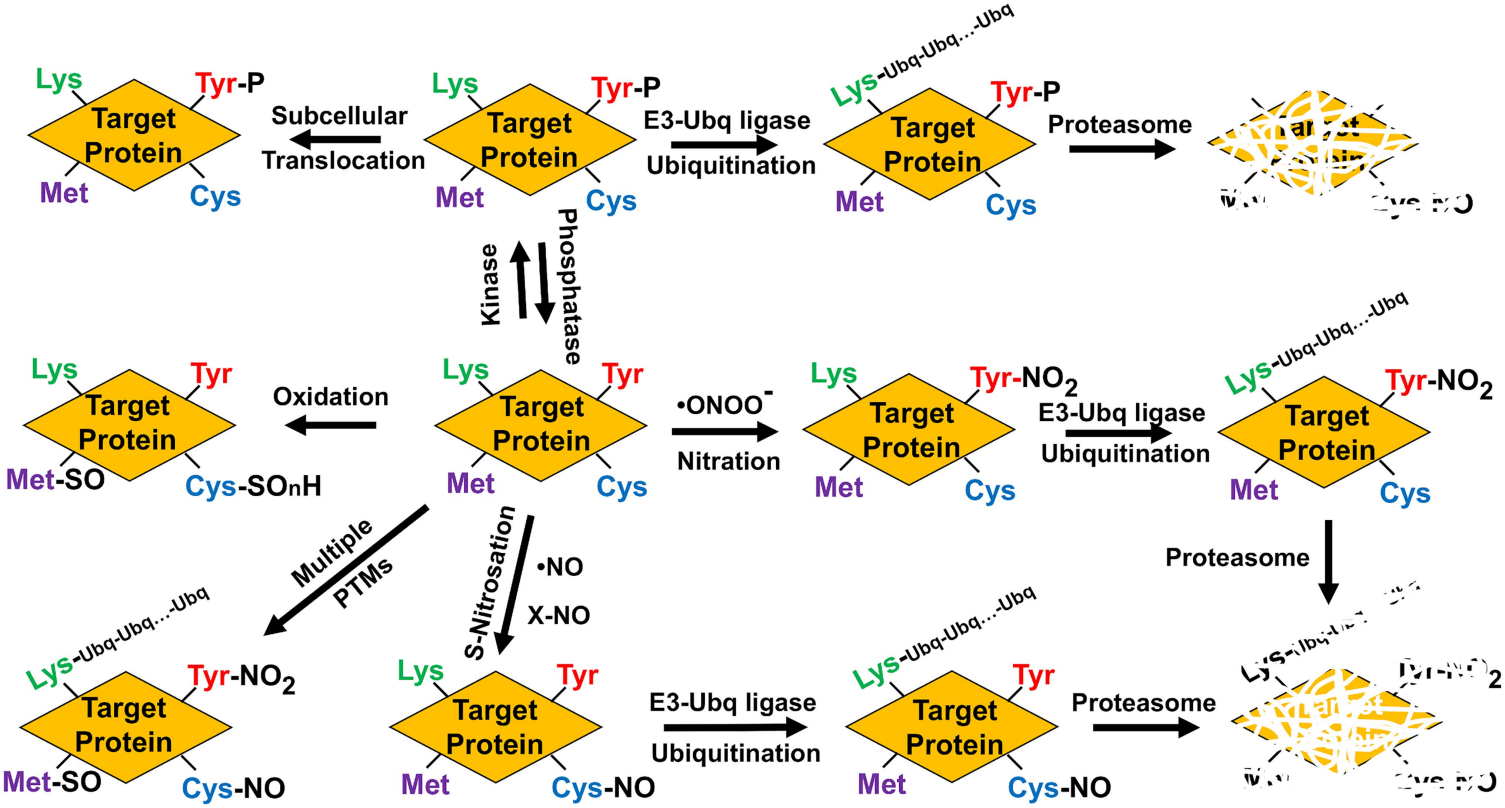
Multiple post-translational modifications potentially alter function/activity, localization, and stability of target proteins. Phosphorylation (P), methionine sulfoxide (Met-SO), Cys oxidation to sulphonic, sulphenic, or sulphinic group (SO_n_H), nitrosoCys (Cys-NO), nitroTyr (Tyr-NO_2_), Ubiquitin (Ubq), and polyubiquitinated lysines (Lys-Ubq-Ubq…-Ubq).

The degradation of nitrated proteins involved in nitrate assimilation and NO production requires an additional PTM as targets should be previously polyubiquitinated in Lys residues before being degraded by the proteasome, therefore connecting nitration with polyubiquitination. NRs are polyubiquitinated (Costa-Broseta et al., 2021) and are translocated to the nucleus by the SUMO E3 ligase SIZ1 (Kim et al., 2018). Remarkably, a recent report showed that proteasomal-mediated cleavage at C-terminal of nitroTyr residues in proteins was reduced when compared to the unmodified residues, making nitration a possible factor that decreases the turnover of oxidized proteins (Ott et al., 2021), and casting doubts on the relationship between protein nitration and proteasome-mediated degradation. However, polyubiquitination is not the only PTM functionally connected to Tyr nitration. Another way Tyr-nitration may have an impact on cell signaling is through interference with a well-known signaling pathway mediated by Tyr-phosphorylation (Gow et al., 1996; Kong et al., 1996). Although Tyr-nitration and Tyr-phosphorylation might be mutually exclusive, some proteins can be simultaneously nitrated and phosphorylated in Tyr residues thus suggesting both PTMs are more competitive than exclusive (Monteiro, 2002; Joshi et al., 2015). Moreover, the nitrating agent peroxynitrite can also act as an inducer of Tyr phosphorylation-mediated signaling events (Takakura et al., 1999; Monteiro, 2002), thus suggesting both PTMs are connected in a complex way. Phosphorylation is very relevant for the regulation of NR and GS activities (Riedel et al., 2001; Lillo et al., 2004), but it occurs in key Ser but not Tyr residues, thus pointing to no phosphorylation-nitration competition in these enzymes. Therefore, protein that are targets of NO-related PTMs can be simultaneously or sequentially modified by other PTMs, which can modify their functions by triggering conformational changes, hampering ligand or cofactor binding, translocating them to different subcellular localization, promoting their preteasome-mediated degradation, or even altering the functional interaction with other signaling pathways (Figure 3).

## Protein Nitration and Metabolism of Reactive Oxygen Species

Stress conditions that rise NO and ABA levels are usually accompanied by ROS such as superoxide anion, hydroxyl radicals, and hydrogen peroxide, which together are involved in multiple signaling pathways (Neill et al., 2002). The levels of ROS are controlled by the activity of antioxidant enzymes, mainly superoxide dismutase (SOD) and catalase (CAT) that metabolize superoxide and hydrogen peroxide, respectively. These enzymes are regulated by NO-related PTMs (Begara-Morales et al., 2016). Nitration and S-nitrosylation of catalases and SODs have been reported (Lozano-Juste et al., 2011; Holzmeister et al., 2015; Begara-Morales et al., 2016; Palma et al., 2020). For *Arabidopsis* SODs, the nitration-mediated inhibition of their activities was specifically exerted in some members of the different families (Holzmeister et al., 2015). Regarding CAT, the enzymes of pepper fruits were inhibited by Tyr nitration during ripening (Chaki et al., 2015), though specific target residues have not yet been identified.

**Figure 4:**
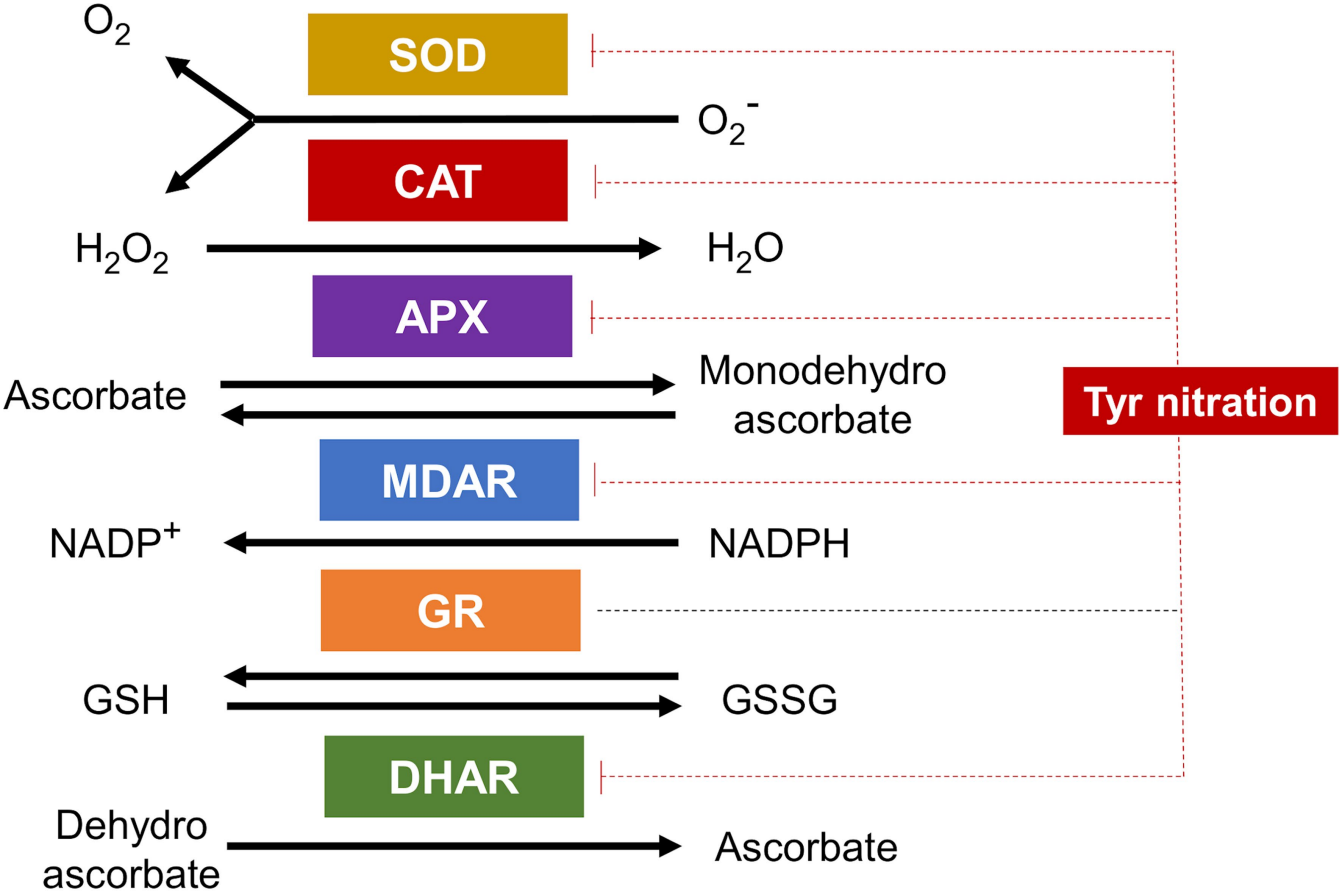
Antioxidant enzymes are targets of Tyr nitration. Superoxide dismutase (SOD), catalase (CAT), ascorbate peroxidase (APX), monodehydroascorbate reductase (MDAR), glutathione reductase (GR), and dehydroascorbate reductase (DHAR) are all targets of Tyr nitration and all but GR inactivated by this PTM (blunt ended red dotted lines).

Hydrogen peroxide levels are also regulated through the so-called ascorbate-glutathione antioxidant cycle involving the activity of ascorbate peroxidases (APX), monodehydroascrobate reductases (MDAR) and dehydroascrobate reductases (DHAR), and glutathione reductases (GR; Noctor and Foyer, 1998). As for SODs and CATs, also enzymes of ascorbate-glutathione cycle have been identified as nitration targets (Lozano-Juste et al., 2011; Begara-Morales et al., 2016). The Tyr nitration sites for pea APX and MDAR have been identified (Begara-Morales et al., 2014, 2015). DHAR and GR have been also identified as Tyr nitration targets in citrus and sunflower, respectively (Chaki et al., 2009; Tanou et al., 2012), but nitration sites remain to be determined. It is worth mentioning that nitration of GR did not affect to their activity (Begara-Morales et al., 2015), thus representing one of the few cases where nitration did not lead to enzyme inactivation. The rest of antioxidant enzymes are inhibited by Tyr nitration thus representing a very relevant node of regulation of ROS metabolism by NO (Figure 4).

## Nitration of Organelle-Located Plant Proteins

A large overrepresentation of protein with organelle localization was identified in the *in vivo* nitrated proteome of *Arabidopsis* that identified 127 nitrated proteins (Lozano-Juste et al., 2011). Around 20, 5, and 3% of the identified nitrated proteins were chloroplastic, mitochondrial, and peroxisomal, respectively (Lozano-Juste et al., 2011). It has been reported that the *Arabidopsis* thylakoid proteome contains more than 100 nitrated proteins (Galetskiy et al., 2011a). Chloroplast metabolism is regulated by Tyr nitration among other PTMs in plastid proteins (Lehtimäki et al., 2015). Photosynthesis-related nitrated proteins involved in the Calvin-Benson cycle have been also identified in *Citrus aurantium* (Tanou et al., 2012). In sunflower and *Arabidopsis*, two key enzymes of photosynthetic carbon metabolism such as glyceraldehyde phosphate dehydrogenase and carbonic anhydrase were inactivated by nitration (Chaki et al., 2009; Lozano-Juste et al., 2011). In addition to central metabolism enzymes, several proteins of the photosystems have been also identified as targets of Tyr nitration (Galetskiy et al., 2011b), which are likely altered in the electron transfer chain and redox signaling. Mitochondria typically contain larger amounts of nitrated proteins with respect to other cellular compartments in different organisms. This is likely due to the high levels of NO and ROS production, thus acting as a continuous source of peroxynitrite (Radi et al., 2002). Peroxisomal enzymes such as serine hydroxymethyl transferase, catalase, alanine:glyoxylate aminotransferase, glycolate oxidase, and malate dehydrogenase have been also identified as nitrated proteins (Lozano-Juste et al., 2011; Sandalio et al., 2019). Some of the identified nitrated plant peroxisome enzymes are involved in reactive oxygen species metabolism thus altering, like above mentioned for chloroplastic proteins, redox signaling (Corpas et al., 2021).

## Genetic Code Expansion and Other Methodologies for the Study of Protein Nitration

Liquid chromatography followed by tandem mass spectrometry (LC–MS/MS) is likely the most used methodology for free and protein-bound 3-nitro-L-tyrosine analyses in biological samples (Larsen et al., 2006; Tsikas and Duncan, 2014). The overwhelming evidence suggesting that the ratio of nitrated vs. non-nitrated protein is usually extremely low likely makes protein nitration identification a very difficult task. It has been calculated that in human plasma the nitroTyr to unmodified Tyr ratio could be around one nitrated molecule per 1 million of non-nitrated molecules (Tsikas and Duncan, 2014). Therefore, the use of enrichment techniques is crucial for the success of subsequent MS-based analysis. For decades, the most widespread methodology for the study of protein nitration has been based on the immunoprecipitation (IP) of proteins with anti-3-nitroTyr antibodies followed by the subsequent identification by LC–MS/MS. These methods have been extensively used in mammals (Zhan and Desiderio, 2009) and plants (Chaki et al., 2009; Lozano-Juste et al., 2011). However, the low proportion of nitrated protein isoforms *in vivo*together with the naturally unstable nature of this PTM made the identification of nitrated protein a challenging task. Because 3-nitro-Tyr are easily reduced to 3-amino-Tyr, MS-based methodologies has required working under strict non-reducing conditions. Unfortunately, it is difficult to ensure that non-reducing conditions are kept during sample manipulation and processing. An alternative methodology is based on the previous full reduction of 3-nitroTyr to 3-aminoTyr. High-Performance Liquid Chromatography (HPLC) technique coupled to electrochemical detection of the N-acetylated, dithionite-reduced derivative of 3-nitroTyr has been proposed for the analysis of nitrated proteins (Shigenaga, 1999). Based on the same principle also the specific enrichment of a targeted nitrotyrosine-containing peptides before MS has been proposed. This method starts by blocking all primary amines by acetylation, reduction of nitroTyr to aminoTyr with dithiothreitol and hemin, and affinity chromatography with an N-hydroxysuccinimide-ester-functionalized stationary phase (Yang, 2017). Another method, using IP with anti-3-nitroTyr followed by reduction of the immunoprecipitated proteins, ligation of biotin tags, streptavidin-based enrichment and final MS detection has been also used (Nikov et al., 2003). The direct MS-based analysis searching for mass shifts of +45 Da in nitrated peptides has been also used to identify nitrated proteins *in vitro* (Lozano-Juste et al., 2011; Medeiros et al., 2021). However, there is abundant evidence pointing to the need of having extreme caution when making assignments based on MS/MS spectra alone, especially when MS/MS resolution or mass accuracy are not good enough. It has been reported an automated method for the validation of endogenous Tyr nitration called rPTMDetermine that enhances the verification through similarity scoring of tandem MS/MS comparisons between modified peptides and their unmodified analogs (Dong et al., 2020). On the other hand, the low levels of nitrated proteins detected *in vivo* has been tried to overcome by exogenous treatment with nitrating agents that largely increase the number and quantity of detected nitrated proteins (Table 1). However, this approach does not allow the identification of proteins that are only nitrated under physiological conditions. Alternatively, genetic strategies based on the use of mutant plants with increased peroxynitrite content should also lead to enhanced levels of protein nitration (Corpas et al., 2009). However, the lack of specificity of nitrating agents such as peroxynitrite leads to the common identification of nitration sites that are not physiologically relevant. This drawback must be solved by using methodologies allowing the specific targeting of nitration events.

**Figure 5:**
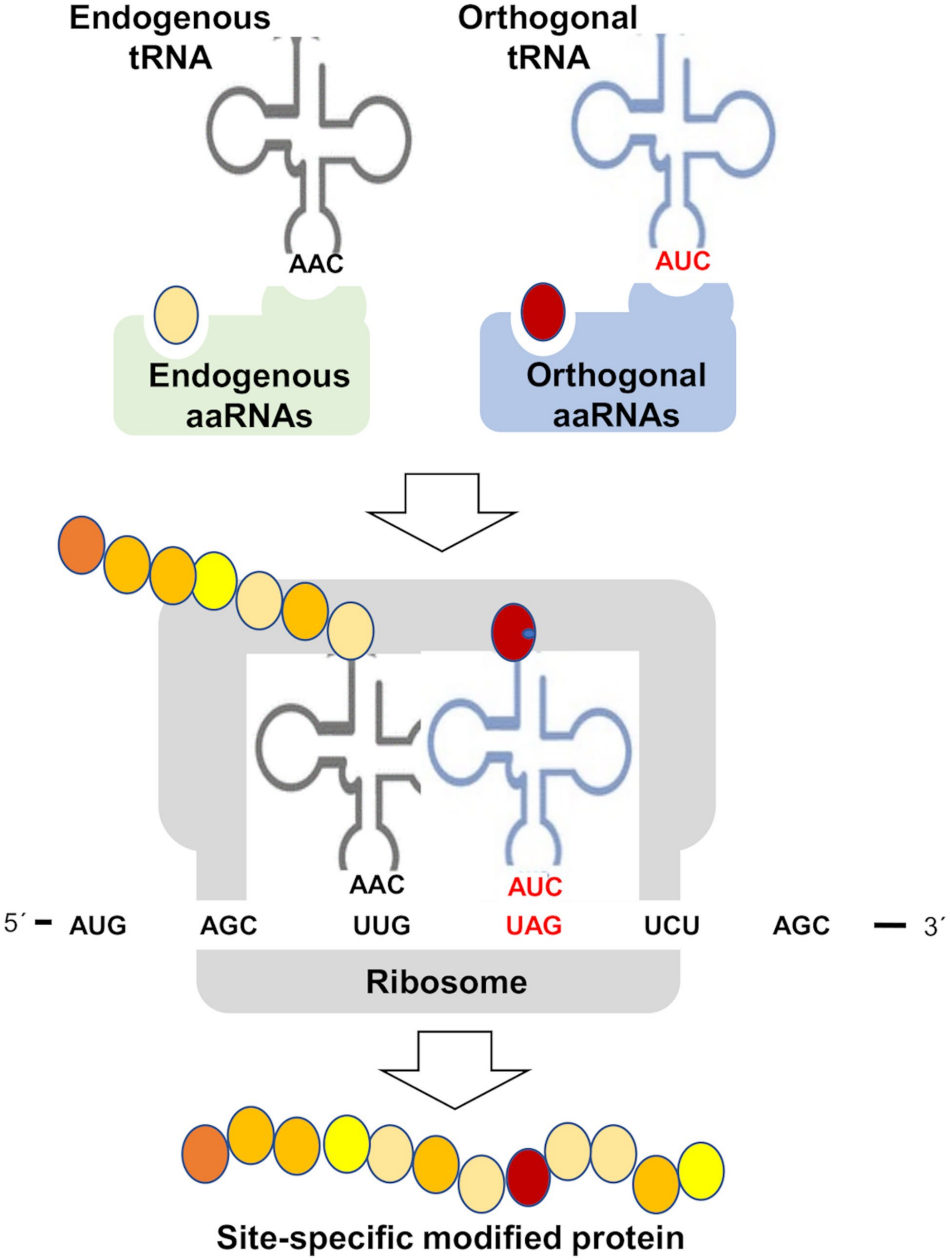
Genetic code expansion methodologies for site-specific incorporation of non-canonical amino acids. Aminoacyl-tRNA synthetase (aaRNAs), transfer RNA (tRNA), and amber stop codon (UAG). This methodology is based on the specificity of aaRNAs/tRNA pairs in such a way that non-canonical amino acid (red circle) and orthogonal tRNA are not substrates of endogenous aaRNAs and vice versa orthogonal aaRNAs do not use canonical amino acids (yellow and orange circles) and endogenous tRNAs. This method generates a site-specific modified protein by incorporation of a non-canonical amino acid, which may be 3-nitroTyr, thus becoming a potentially useful tool to study Tyr nitration or any other PTM specific effects on target proteins.

Likely the most used specific methodology to study the effects of PTMs on protein function has been the site-specific mutagenesis. In most of the cases, the strategies were based on substitution of amino acids for others that mimic a specific PTM. Ser and Thr change to Glu or Asp has been used to mimic phosphorylation. However, this approach just allows imitating the changes in charge and steric effects caused by PTMs, but it is often far from the real PTM. A potential alternative would be the chemical synthesis of PTM-related functional groups into the protein backbone, a procedure that allows site-specific modifications of a desired protein and afford the product in large quantities for biochemical and structural analyses (Siman and Brik, 2012). This technique has been used for glycosylation, phosphorylation, ubiquitination, acetylation, and lipidation (Siman and Brik, 2012), but to our knowledge nor for nitration of S-nitrosylation. In any case, this strategy relies on the stability of the modified protein and on the chemistry used for synthesis that commonly involves the thiol group of Cys residues, which is the target residue for S-nitrosylation. An alternative method is based on the capacity of bacteria to perform genetically encoded synthesis of proteins containing non-proteinogenic amino acids including 3-nitroTyr (Beyer et al., 2020; Jang et al., 2020; Chen and Tsai, 2021), therefore representing a valuable platform for studying specific protein nitration effects. These genetic code expansion methodologies allow the co-translational incorporation of non-canonical amino acids into proteins (Figure 5). The method requires de expression of an orthogonal aminoacyl-tRNA synthetase/t-RNA pair for the modified amino acid of interest. The resulting aminoacylated orthogonal tRNA is used by ribosomes to decode and amber stop codon at the position, where the non-proteinogenic amino acid should be incorporated (Nödling et al., 2019). This genetic code expansion technologies are useful for incorporating nitroTyr residues to proteins by using 3-nitroTyr tRNA synthetases (Cooley et al., 2014; Beyer et al., 2020). Most of the uses of orthogonal aminoacyl-tRNA synthetase/t-RNA pairs have been reported for mammal cells. By using this genetically encoding 3-nitro-tyrosine into the tyrosine nitration sites of human indoleamine 2,3-dioxygenase 1 and α-synuclein, the native nitrated proteins have been obtained and used for assessing the effects of nitration of different Tyr sites on the structure, function, and enzyme activity (Gerding et al., 2019; Zheng et al., 2020). Site-specific nitration of calmodulin at its two Tyr residues using genetic code expansion technology allowed assessing the effects of these alterations on calcium binding by calmodulin, and on the subsequent binding and activation of human endothelial NOS (eNOS; Porter et al., 2020). By substituting a key Tyr residue by nitroTyr using genetic code expansion technologies, the primary electron transfer in the reaction centers of the photosynthetic bacteria *Rhodobacter sphaeroides* has been studied and further engineered (Weaver et al., 2021). This genetically encoded synthesis of proteins containing 3-nitroTyr residues opens a potentially valuable strategy to assess the functional relevance of site-specific nitration on diverse target proteins, but to date no such application has been reported for any plant protein.

**Table 1:** Reported identification of nitrated Tyr residues of plant proteins.

Protein	Organism	Nitrated Tyr residues	Functional site	Effect	References
Nitrate reductase (NIA1)	*Arabidopsis*	714	FAD-binding	−	Costa-Broseta et al. (2021)
Nitrate reductase (NIA2)	*Arabidopsis*	733	FAD-binding	−	Costa-Broseta et al. (2021)
Cyclin-dependent kinase A (CDKA)	Maize	15, 19	ATP-binding	−	Méndez et al. (2020)
Nitrite reductase 1 (NiR1)	*Arabidopsis*	147, 155, 414, 553	Out of catalytic pocket	−	Costa-Broseta et al. (2020)
NADP-malic enzyme 2	*Arabidopsis*	73	Dimer and tetramer interface	−	Begara-Morales et al. (2019)
Pollen allergen (Bet v 1a)	Birch	5, 66	Not defined		Gusenkov and Stutz (2018)
Thaumatin-like protein E2	Tobacco	36	Not defined		Takahashi et al. (2016)
ABA receptor PYR1	*Arabidopsis*	58, 120	ABA-binding pocket	−	Castillo et al. (2015)
ABA receptor PYL4	*Arabidopsis*	80	ABA-binding pocket	−	Castillo et al. (2015)
ABA receptor PYL8	*Arabidopsis*	60, 158	ABA-binding pocket	−	Castillo et al. (2015)
PS II oxygen-evolving complex 1 (PsbO1)	*Arabidopsis*	9			Takahashi et al. (2015)
Monodehydroascorbate reductase (MDAR)	Pea	213, 292, 345	NADP-binding	−	Begara-Morales et al. (2015)
Mitochondrial manganese superoxide dismutase (MSD1)	*Arabidopsis*	63	Substrate binding	−	Holzmeister et al. (2015)
Leghemoglobin		130	Distal heme pocket		Sainz et al. (2015)
Pollen allergen Bet v 1.0101	Birch	81, 83, 150, 158	Hydrophobic cavity and C-terminus		Reinmuth-Selzle et al. (2014)
Ascorbate peroxidase (APX)	Pea	5, 235	Heem group pocket	−	Begara-Morales et al. (2014)
NADP-isocitrate dehydrogenase	Pea	392	NADP^+^ binding		Begara-Morales et al. (2013)
NADH-dependent hydroxypyruvate reductase1 (HPR1)	*Arabidopsis*	97, 108, 198	Active site	−	Corpas et al. (2013)
Glutamine synthetase (GS)	*Medicago truncatula*	167	Not defined	−	Melo et al. (2011)
O-acetylserine(thiol) lyase (OASA1)	*Arabidopsis*	302	Pyridoxal-5′-phosphate binding	−	Alvarez et al. (2011)
PSII complex protein PSBA	*Arabidopsis*	237, 262		−	Galetskiy et al. (2011a,b)
Methionine synthase	*Arabidopsis*	287	Not defined	−	Lozano-Juste et al. (2011)
Plastocyanin	Spinach	83	Cofactor binding	+	Anderson et al. (1985)

Besides MS-based methods, a wide array of chromatographic, immunochemical, and bio-sensing techniques has been reported to be useful for the identification and quantification of protein Tyr-nitration (recently reviewed in Bandookwala et al., 2020). After HPLC, nitrated proteins have been quantified also by UV detection (Yang et al., 2010); by electrochemical detection with sensitivity and selectivity compared to immunodetection methods (Vujacic-Mirski et al., 2020); by fluorescence detection after derivatization (Pourfarzam et al., 2013); and also by photodiode array detection (Teixeira et al., 2017). Size exclusion chromatography coupled to reverse phase-HPLC with diode array detection has been also developed to evaluate protein nitration (Liu et al., 2017). Nitrated protein identification has benefited also from other spectroscopic techniques. It has been recently reported the use of Surface enhanced Raman spectroscopy (SERS) to identify some 3-nitroTyr-containing neurodegenerative disease-related proteins that are converted to azobenzene containing peptides using a protocol based on silver nanoparticles stabilized by citrate (Niederhafner et al., 2021). Protein Tyr nitration has been also detected by electron paramagnetic resonance (EPR) spin trapping (Romero et al., 2003; Chen et al., 2004).

## Concluding Remarks and Perspectives

The identification of nitrated proteins and the involvement of this PTM on the regulation of diverse plant physiological processes has gained increasing interest in the last decades. However, much more work will be needed to fill the multiple gaps remain in our knowledge of the regulatory functions exerted by Tyr protein nitration in plants. The low proportion of nitrated peptides in the *in vivo* proteomic analyses together with its inherent instability make the identification of nitration sites a difficult task. The use of artificial nitration procedures is likely not the best strategy for nitration site identification due to the lack of specificity of nitrating systems. Therefore, more work must be performed on the application of genetic code expansion technologies based on the use of orthogonal aminoacyl-tRNA synthetase/tRNA pairs engineered for specific non-canonical amino acids. These methodologies permit the programmable site-specific installation of genetically encoded 3-NT sites in proteins expressed in *Escherichia coli* thus assessing the effects of specific nitration. These recombinant proteins containing PTMs will represent a very valuable tool for providing insights into how specific modifications regulate protein structure and function. Genetic code expansion methodologies will open not only the possibility to work with bacteria-expressed site-specific modified recombinant proteins *in vitro*, but also will allow to engineer plants by expressing the orthogonal aRNAs/tRNA pairs and supplying the non-canonical amino acid of interest, thus allowing the expression of specifically modified protein versions with agro-biotechnological advantages. However, this strategy will require significant advances on our knowledge of these processes and also to explore the feasibility of application for plants. In any case, a deeper knowledge on the function of Tyr nitration of plant proteins will certainly help to understand the functional relevance of this PTM not only in stress-related conditions, when this modification is more prevalent, but also under non-stress conditions, when nitrated targets may play or stop playing decisive regulatory roles on growth and development. Therefore, better known Tyr nitration targets and mechanisms should allow also a deeper knowledge on the signaling nodes involved in controlling plant physiology.

## Author Contributions

JL compiled and organized the reported information and wrote the article.

## Funding

The work on hypoxia in the JL laboratory is supported by grants BIO2017-82945-P and PID2020-112618GBI00 funded by MCIN/AEI/10.13039/501100011033 and by the European Union.

## Conflict of Interest

The author declares that the research was conducted in the absence of any commercial or financial relationships that could be construed as a potential conflict of interest.

## Publisher’s Note

All claims expressed in this article are solely those of the authors and do not necessarily represent those of their affiliated organizations, or those of the publisher, the editors and the reviewers. Any product that may be evaluated in this article, or claim that may be made by its manufacturer, is not guaranteed or endorsed by the publisher.
